# The Rare Case Presentation of Adult-Onset Fulminant Subacute Sclerosing Panencephalitis in a 24-Year-Old Male

**DOI:** 10.7759/cureus.68985

**Published:** 2024-09-09

**Authors:** Nikhil Malunjkar, Pradeep Tiwari, Neha Momale, Vijayalaxmi Pujari, Smita Patil, Arundhati Barua, Tejaswee Lohakare

**Affiliations:** 1 General Medicine, Padmashree Dr. D.Y. Patil School of Medicine, Nerul, IND; 2 Child Health Nursing, Smt. Radhikabai Meghe Memorial College of Nursing, Wardha, IND

**Keywords:** brain mri flair signals, generalized tonic-clonic seizures, intrathecal interferon-α (ifn-α), measles antibody titer, subacute sclerosing panencephalitis (sspe)

## Abstract

Subacute sclerosing panencephalitis (SSPE) is a late effect of measles in children. Its features include seizures, a gradual loss of physical and cognitive function, and finally death. Despite the absence of a definitive cure for this disorder, a regimen combining intrathecal interferon-α (IFN-α) and daily oral isoprinosine has demonstrated effectiveness. We present the case of a 24-year-old male with spastic seizure epilepsy. He exhibited progressive weakness, frequent postural instability, and recurrent generalized tonic-clonic seizures. Increased measles antibody concentrations in the cerebrospinal fluid (CSF), prominent amplitude spikes on the electroencephalogram (EEG), and heightened fluid-attenuated inversion recovery (FLAIR) signals on brain magnetic resonance imaging (MRI) suggested a diagnosis of SSPE.

## Introduction

The symptoms of subacute sclerosing panencephalitis (SSPE) include seizures, a gradual decline in cognitive and physical abilities, and death that happens five to 15 years after the measles virus infection. A person who contracted measles when younger than two years old is most likely to develop SSPE [1]. While measles virus infection is frequent in the Middle East and India, it is comparatively uncommon in the Western world [2,3]. In underdeveloped nations, there are 10-20 cases of SSPE per million people annually [4,5]. Due to immune system immaturity, the chance of developing SSPE increases with earlier age of measles virus exposure before symptoms become apparent [6]. The measles virus has the capability to lie dormant within cells for multiple years. Ultimately, the virus induces an inflammatory response directed at the affected cells, causing glial scarring and elevated astrocyte growth, lymphocyte and infiltration of plasma cells, neuronal damage, and loss of myelin [4,7,8]. A distinctive electroencephalogram (EEG) pattern and an elevated concentration of anti-measles IgG antibodies in the serum and cerebrospinal fluid (CSF) are used to make the diagnosis. Between four and 22 years is the mean average latent time from measles to SSPE [2,6]. There have only been a relatively small number of adult-onset SSPE cases previously reported [2,6,7].

## Case presentation

**Figure 1:**
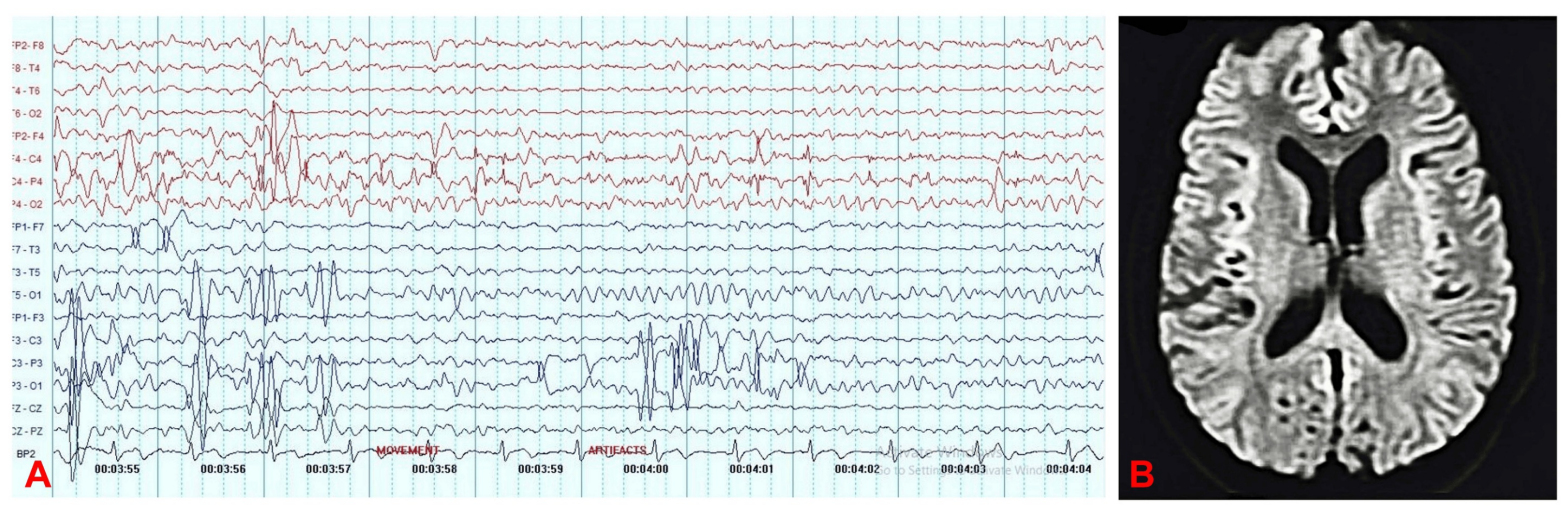
(A) Electroencephalogram (EEG) and (B) magnetic resonance imaging (MRI) brain 1(A) displays an electroencephalogram (EEG), indicating generalized slowing characterized by theta waves. 1(B) presents a contrast-enhanced magnetic resonance imaging (MRI) scan of the brain, revealing effacement of the sulcal spaces in the bilateral parietal, temporal, and occipital regions, along with subtle leptomeningeal enhancement on the post-contrast images, which is suggestive of meningitis.

A 24-year-old male was admitted to our hospital with myoclonic jerks for the past three days, altered sensorium, and generalized weakness for the past one day. Three days ago, he experienced a sudden onset of myoclonic jerks associated with loss of postural control and a fall as his initial symptoms. At that time, the abnormal body movements went unnoticed by his relatives at home. Subsequently, he experienced a generalized tonic-clonic seizure event. Unable to carry out daily activities, he was brought to our center. At our center, the patient received antiepileptic medication. Upon presentation, the patient was vitally stable with hyperreflexia and hypertonia. However, despite the medication, the myoclonic jerks persisted. CSF studies were unremarkable, and all blood investigations were within normal range. EEG showed generalized slowing with theta waves, and magnetic resonance imaging (MRI) of the brain with post-contrast (P+C) revealed effacement of the sulcal spaces in the bilateral parietal, temporal, and occipital regions, along with subtle leptomeningeal enhancement, suggestive of meningitis (Figure 1). The autoimmune encephalitis panel was negative. During his hospital stay, he experienced multiple brief bouts of abrupt generalized weakness and loss of postural stability. His condition steadily deteriorated as these incidents became more frequent and his weakness worsened. A neurological examination after two weeks of hospital stay found that the paralysis had become flaccid. A rare possibility of SSPE was considered, as an elevated titer of 625 IgG anti-measles antibodies was detected in the CSF by enzyme-linked immunosorbent assay (ELISA).

## Discussion

SSPE is an extremely rare and delayed measles consequence. The majority of children with SSPE typically have a history of measles infection occurring before the age of two [9]. Our patient, who acquired measles at two months old, exhibited worsening weakness and numerous episodes of tonic-clonic seizures, despite myoclonic seizures being more commonly linked with SSPE. Individuals with SSPE frequently exhibit behavioral issues, deterioration in cognition, sudden involuntary jerks, convulsions, and visual disturbances. Patients sometimes have pyramidal and extra-pyramidal indications as the condition worsens. Their myoclonus may go away, they become quadriplegic, and their spasticity worsens. Later phases may see the development of decerebrate and decorticate posture, as well as loud and irregular breathing [9-11]. It ends with a chronic vegetative state, and death usually happens in a year or three [9]. Based on the clinical presentation, the disease's course can be rated [10,11]. Normal cellular and biochemical signs are usually seen during CSF investigation, in addition to elevated protein levels. Anti-measles antibody titers are persistently high in serum and CSF, despite the absence of the distinctive oligoclonal pattern. Serum and CSF levels of anti-measles antibodies 1:256 or above, respectively, are considered elevated and suggestive of SSPE [9].

It could be a normal early EEG. The EEG pattern might display unique cyclical waveforms, first identified by Cobb et al. [12], as the illness worsens. Irregular periodic complexes are linked to myoclonic seizures. In later phases, the distinctive periodic complexes vanish and the EEG becomes increasingly disorganized, as was the case with our patient. Regarding neuroimaging, abnormalities of the white matter and basal ganglia are believed to be easier to see on an MRI [13]. In the periventricular region, our patient additionally exhibited extensive areas of elevated T2-weighted/fluid-attenuated inversion recovery (FLAIR) signals, which is in line with research indicating that periventricular and the sub-cortical white matter are the areas most often impacted [9]. In the latter stage, brainstem, cerebellum, and hemisphere regions show progressive atrophy according to neuroimaging [9]. Confirming the diagnosis of SSPE requires a brain when typical clinical and EEG symptoms are not present. The polymerase chain reaction (PCR) is the most effective method for detecting the ribonucleic acid (RNA) genomes of the measles virus in brain tissue. In our case, resource limitations prevented us from doing a brain biopsy.

The diagnosis of SSPE is established through characteristic clinical signs, recurrent EEG complexes, and increased measles antibody levels in the CSF. Based on Dyken's diagnostic criteria, a diagnosis is deemed probable if a patient satisfies three out of the five criteria and definitive if the patient meets the fifth criterion as well [9]. Our patient is classified as probable. We have ruled out a number of significant differential diagnoses associated with SSPE, such as brain tumors, encephalitis, Wilson's disease, Huntington's disease, and cerebral venous thrombosis (CVT). Psychiatric disorders such as malingering, schizophrenia, and depression were also disregarded. This illness has no known cure. The optimum outcome for symptomatic management has been reported to be a combination of daily oral isoprinosine and weekly intrathecal IFN-α [4].

A rare and deadly side effect of paediatric measles is SSPE. It is an incurable, progressive illness that usually takes one to three years to fully manifest symptoms. The spectrum of clinical presentation is broad, encompassing symptoms such as seizures, extrapyramidal and pyramidal symptoms, increasing weakening, and coma. To gain a better understanding of the natural history and suitable management of this illness, more research and analysis are necessary. Acute fulminant SSPE has an atypical presentation, with progressive worsening of symptoms; hence, it has become a diagnostic challenge. Risk factors for fulminant SSPE are previous measles virus infection, impaired host defense mechanisms, and immune-compromised conditions. Patients with fulminant SSPE seldom meet Dyken's clinical and EEG criteria; hence, a high level of suspicion is needed to rule out this possibility. There are few therapeutic options available for SSPE, and nearly all patients have a fatal result. Most patients pass away within one to three years of being sick.

## Conclusions

SSPE is a fatal disease of the central nervous system (CNS). With strong clinical suspicion, anti-measles antibodies should be included in investigations. Treatment available is very costly, and in very few patients showed prolonged some part of life. Hence, vaccination is a must in children and females of childbearing age.
